# Correction: Genetically tunable frustration controls allostery in an intrinsically disordered transcription factor

**DOI:** 10.7554/eLife.35768

**Published:** 2018-02-12

**Authors:** Jing Li, Jordan T White, Harry Saavedra, James O Wrabl, Hesam N Motlagh, Kaixian Liu, James Sowers, Trina A Schroer, E Brad Thompson, Vincent J Hilser

Li J, White JT, Saavedra H, Wrabl JO, Motlagh HN, Liu K, Sowers J, Schroer TA, Thompson EB, Hilser VJ. 2017. Genetically tunable frustration controls allostery in an intrinsically disordered transcription factor. *eLife* **6**:e30688. doi: 10.7554/eLife.30688.

Published 12 October 2017

In one of our figures and its supplement (namely Figure 2A and B, and Figure 2—Figure supplement 1B), we compared new unpublished data with data that had previously been published. Although reference is made to this fact in the text and in the legends, differences between the current manuscript and Li, et al. 2012, regarding the nomenclature used to describe identical samples, requires additional clarification. As such, we have provided additional text for the corresponding legends.

The following text has been added to the legend for Figure 2: ‘We note that data for the R-F and F domains in panels (a) and (b) are the same as presented in Li et al., 2012 to allow for direct comparison of the opposing effects of the DBD and the R-domain on the F-domain.’

Similarly, the following text has been added to the legend of Figure 2—Figure supplement 1: ‘We note that the data in (b) are the same as presented in Li et al. 2012 (Fig. 4a), according to the current nomenclature, wherein the R-F construct is equivalent to the A isoform of NTD and the F construct is equivalent to the C3 isoform of NTD.’

The original Figure 2 legend is shown here for reference:

Coupling of the R-domain and DBD to the functional F-domain in GR.

(a) TMAO-induced folding for the F-domain alone and with either the R-domain or the DBD. (b) Protease sensitivity assay: comparing F-domain and F-domain with R-domain (left) performed at a protein (1 mg/ml): trypsin mass ratio of 1000:1 (Li et al., 2012); comparing F-domain and F-domain with DBD (right) performed at a protein (1 mg/ml): trypsin mass ratio of 100:1. Note: each protein pair (i.e. F vs. R-F and F vs. F-DBD) was run on the same gel, with the intervening lanes removed in the figure for clarity. (c) Luciferase assay for C3 isoform (GR F-DBD) versus chimeric construct (GR F-Gal4 DBD).

The corrected Figure 2 legend follows:

Coupling of the R-domain and DBD to the functional F-domain in GR.

(a) TMAO-induced folding for the F-domain alone and with either the R-domain or the DBD. (b) Protease sensitivity assay: comparing F-domain and F-domain with R-domain (left) performed at a protein (1 mg/ml): trypsin mass ratio of 1000:1 (Li et al., 2012); comparing F-domain and F-domain with DBD (right) performed at a protein (1 mg/ml): trypsin mass ratio of 100:1. Note: each protein pair (i.e. F vs. R-F and F vs. F-DBD) was run on the same gel, with the intervening lanes removed in the figure for clarity. (c) Luciferase assay for C3 isoform (GR F-DBD) versus chimeric construct (GR F-Gal4 DBD). We note that data for the R-F and F domains in panels and (b) are the same as presented in Li et al., 2012 to allow for direct comparison of the opposing effects of the DBD and the R-domain on the F-domain.’

The original Figure 2—Figure supplement 1 legend is shown here for reference:

Domain stabilities determined by TMAO-induced protein folding transitions.

(a) Thermodynamic parameters obtained from TMAO-induced folding experiments demonstrate that DBD stabilizes a folded conformation of the F-domain while the R-domain destabilizes that conformation. Parameter values obtained from the fits of the TMAO-induced folding data for the constructs shown in Figure 2a. See previous publication (Li et al., 2012) for details. Because the thermodynamic analyses report on the free energy differences and not the mechanistic bases of the energy differences, the reported free energies of folding (ΔG0F−UΔGF−U0) may not necessarily reflect the stability of unique conformations. Instead, they may be reporting on the stability of a conformationally heterogeneous ensemble. Toward this end, it has been reported that ID proteins may adopt poly-beta sheet formations (Han et al., 2012) as part of their functional states. As the effect of TMAO on structure is almost entirely defined by the influence on backbone atoms (i.e. excluding backbone hydrogen bond donors and acceptors from solvent) (Auton and Bolen, 2005), any state or ensemble of states that facilitates removal of backbone atoms from solvent will be stabilized by TMAO. As such, the current analysis provides evidence that a functional state exists whose probability is proportional to transcriptional activation. However, the structural properties of that state are not known. (**b**) Comparison of the *m-values* fitted from TMAO induced folding of F-domain and R-F with the published *m-values* for known representative globular proteins (see previous publication (Li et al., 2012) for details). The *m-value* comparison suggests that the F-domain and R-domain can adopt folded conformations similar to globular proteins in terms of surface area buried upon folding.

The corrected Figure 2—Figure supplement 1 follows:

a) Thermodynamic parameters obtained from TMAO-induced folding experiments demonstrate that DBD stabilizes a folded conformation of the F-domain while the R-domain destabilizes that conformation.

Parameter values obtained from the fits of the TMAO-induced folding data for the constructs shown in Figure 2a. See previous publication (Li et al., 2012) for details. Because the thermodynamic analyses report on the free energy differences and not the mechanistic bases of the energy differences, the reported free energies of folding (ΔG0F−UΔGF−U0) may not necessarily reflect the stability of unique conformations. Instead, they may be reporting on the stability of a conformationally heterogeneous ensemble. Toward this end, it has been reported that ID proteins may adopt poly-beta sheet formations (Han et al., 2012) as part of their functional states. As the effect of TMAO on structure is almost entirely defined by the influence on backbone atoms (i.e. excluding backbone hydrogen bond donors and acceptors from solvent) (Auton and Bolen, 2005), any state or ensemble of states that facilitates removal of backbone atoms from solvent will be stabilized by TMAO. As such, the current analysis provides evidence that a functional state exists whose probability is proportional to transcriptional activation. However, the structural properties of that state are not known. (b) Comparison of the *m-values* fitted from TMAO induced folding of F-domain and R-F with the published *m-values* for known representative globular proteins (see previous publication (Li et al., 2012) for details). The *m-value* comparison suggests that the F-domain and R-domain can adopt folded conformations similar to globular proteins in terms of surface area buried upon folding. We note that the data in (b) are the same as presented in Li et al. 2012 (Fig. 4a), according to the current nomenclature, wherein the R-F construct is equivalent to the A isoform of NTD and the F construct is equivalent to the C3 isoform of NTD.

The article has been corrected accordingly.

